# *Sirt2* ablation exacerbates *Sod1* knockout-induced progeroid phenotype in mice

**DOI:** 10.1016/j.redox.2025.103770

**Published:** 2025-07-15

**Authors:** Anke Geng, Xiaona Wang, Zhenkai Wu, Zhihao Liu, Xiao Huang, Xiyue Wang, Xiaoxiang Sun, Yingjie Wang, Jiayu Chen, Ying Jiang, Huanyin Tang, Zhiyong Mao

**Affiliations:** a Shanghai Key Laboratory of Maternal Fetal Medicine, Clinical and Translational Research Center of Shanghai First Maternity and Infant Hospital, Frontier Science Center for Stem Cell Research, School of Life Sciences and Technology, Tongji University, Shanghai, 200092, China; b School of Medicine, Tongji University, Shanghai, 200092, China; c Shanghai Key Laboratory of Signaling and Disease Research, School of Life Sciences and Technology, Tongji University, Shanghai, 200092, China

**Keywords:** Genomic stability, Progeroid, Oxidative stress, *Sirt2*

## Abstract

The free radical theory of aging suggests that oxidative stress from free radicals contributes to aging. While free radicals cause DNA damage and cellular dysfunction, they also regulate essential signaling pathways. This duality complicates direct testing of the theory in mice, as demonstrated by the minor lifespan impact of *Sod1* knockout. We hypothesize that base excision repair mechanisms, that involve SIRT2, a longevity-associated SIRTUIN family member, may mitigate excessive free radical effects. Our study found that *Sirt2*^*−/−*^*Sod1*^*−/−*^ double-knockout mice exhibited significantly reduced lifespan and progeroid phenotypes, including spinal curvature and tissue degeneration. These mice displayed increased aging-related gene expression, cellular senescence, enlarged spleens, elevated cytokines, and immune dysregulation, potentially leading to cytokine storm-related deaths. Additionally, *Sirt2* overexpression rescued genomic instability caused by *Sod1* deficiency in cells. These findings refine free radical theory of aging and highlight SIRT2 as a target for enhancing DNA repair and mitigating aging-associated phenotypes.

## 1 Introduction

In 1956, Denham Harman proposed the free radical theory of aging, which posits that the aging process is fundamentally driven by the accumulation of oxidative stress due to free radicals generated during aerobic respiration [1]. This theory suggests that the intrinsic process of aging is caused by cumulative oxidative damage to cells. Numerous studies have indeed demonstrated that the levels of free radicals increase with age across different species as reviewed in Refs. [[2], [3], [4], [5]], potentially leading to DNA damage and genomic instability, which in turn can cause cellular senescence.

However, the relationship between free radical scavengers and aging in mice is complex. For instance, mice lacking *Nrf2*, a key regulator of cellular antioxidant response and detoxification pathways, show little to no effect on aging phenotype median lifespan [[6], [7], [8]]. Similarly, mice deficient in *Sod1*, an enzyme crucial for scavenging superoxide radicals, only experience an approximately 30 % reduction in lifespan [9]. In the case of catalase-deficient mice, premature aging phenotypes are observed, but these are attributed more to metabolic changes than to the accumulation of free radicals [10]. These *in vivo* studies have sparked debate and further investigation into the role of free radicals in aging, suggesting that the relationship between free radical accumulation and aging may not be as straightforward as initially proposed by the free radical theory of aging. The complexity of free radicals in cellular biology is underscored by their dual role as integral components of cellular signaling pathways [[11], [12], [13]]. Excessive elimination of free radicals can lead to cellular dysfunction and the disruption of tissue homeostasis. Indeed, transgenic mice overexpressing *Sod1* do not exhibit extended lifespans or changes in aging phenotypes; on the contrary, their lifespans are shortened [14]. Another potential reason we do not observe the phenotype of severely accelerated aging due to free radical accumulation is that after inflicting oxidative damage to macromolecules such as DNA, cells possess a secondary defense mechanism involving base excision repair (BER) to maintain normal cellular functions [15], thereby maintaining tissue homeostasis and preventing the emergence of aging phenotypes. Providing *in vivo* data on the accelerated aging of animals with simultaneous knockout of key genes involved in free radical scavenging, such as *Sod1*, and genes regulating BER pathway will help to substantiate the free radical theory of aging and lay the foundation for the development of new intervention strategies.

The BER pathway is a vital mechanism for repairing minor DNA base damage induced by free radicals caused oxidative stress, chemical modifications, or spontaneous hydrolysis [16]. The process begins with DNA glycosylases, such as OGG1 (8-oxoguanine glycosylase), which identifies and excises the damaged base [17]. Following this, the enzyme APE1 (apurinic/apyrimidinic endonuclease) acts as an AP endonuclease, cutting the DNA backbone at the abasic site left by the glycosylase [18]. Subsequently, the DNA polymerase β or other polymerases fill in the missing base, aligning with the undamaged complementary strand [19,20]. Lastly, the DNA ligase complex seals the remaining nick, thereby finalizing the repair and preserving the genome integrity, which is crucial for preventing mutations [[21], [22], [23]]. Knocking out critical BER factors including *Ape1*, *Xrcc1* or DNA polymerase β all leads to early-stage embryonic or postnatal lethality [[24], [25], [26], [27]], indicating that it is probably not a feasible approach to simultaneously knock out *Sod1* and these essential BER factors to validate the free radical theory of aging.

Our earlier research has shown that SIRT2, one member of the Sirtuin protein family, modulates the BER pathway by enhancing the binding of OGG1 to its promoter, thereby upregulating its expression [28]. This regulatory mechanism is contingent upon stress, as the DNA damage sensors ATM and ATR trigger the role of SIRT2 role in BER. This indicates that SIRT2 is activated to modulate BER specifically under conditions of oxidative stress-induced DNA damage. Significantly, mice deficient in *Sirt2* have not been reported to exhibit alterations in aging phenotypes, which provides us with the opportunity to investigate the combined impact of free radical accumulation and partial BER deficiency on aging phenotypes in mice.

In this study, we found that mice lacking both *Sirt2* and *Sod1* had a reduced lifespan and showed severe premature aging features, including spinal curvature and skin ulcers. These mice had higher levels of aging-related gene expression and cellular senescence in various organs compared to controls. They also displayed signs of inflammation and immune cell activation, possibly leading to a cytokine storm and early death. *Sirt2* overexpression mitigated the genomic instability and senescence effects caused by the absence of *Sod1* in mouse cells. This indicates that the loss of both antioxidant defense and DNA repair accelerates aging, reinforcing the free radical theory of aging, and positions SIRT2 as a potential target for anti-aging interventions.

## 2 Results

### 2.1 The absence of *Sirt2* exacerbates the aging phenotypes in *Sod1* knockout mice

To further validate the free radical theory of aging, we generated double knockout (DKO, *Sirt2*^*−/−*^*Sod1*^*−/−*^) mice by crossing mice lacking *Sirt2*, which responds to oxidative damage and is involved in regulating BER [28], with *Sod1* knockout mice. Western blot analysis using the protein extracts from brains of different groups of mice confirmed the successful breeding (Supplementary Fig. 1A). Using these DKO mice, along with separate cohorts of wild-type (WT), *Sirt2* knockout (*Sirt2*^*−/−*^), and *Sod1* knockout (*Sod1*^*−/−*^) mice, we conducted subsequent analysis on lifespan and aging-related phenotypes.

**Fig. 1:**
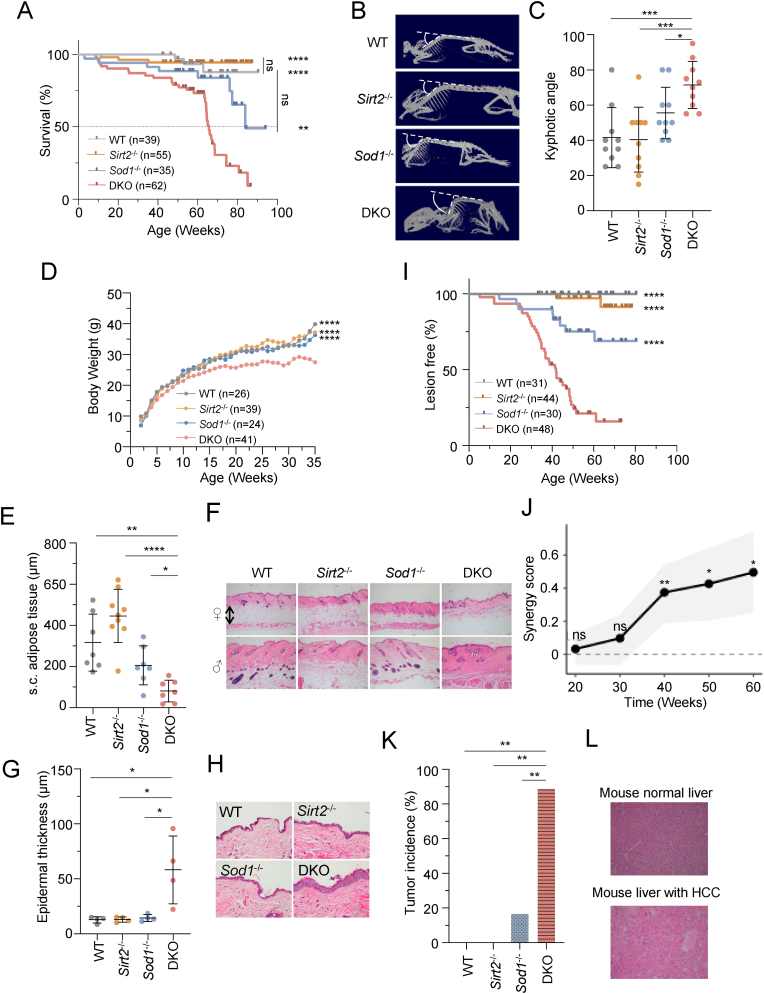
Exacerbated aging phenotypes in *Sod1*^*−/−*^*Sirt2*^*−/−*^ mice in comparison to *Sod1*^*−/−*^ or *Sirt2*^*−/−*^ mice. (A) Kaplan-Meier survival curves of mice of the indicated genotypes over a 1.63-year period. n = 35–62 mice per genotype. (B) Representative X-ray images of 12–14-month-old mice are shown. White circle arc indicates curvature angle measurements. (C) Kyphosis analysis of angle measurements in B. n = 10 mice per genotype. (D) Body weight curves for WT, *Sirt2*^*−/−*^*, Sod1*^*−/−*^ and DKO mice. n = 24–41 mice per genotype. (E) Subcutaneous (s.c.) fat thickness in 12–14-month-old DKO mice compared to 12-14-month-old WT, *Sirt2*^*−/−*^, *Sod1*^*−/−*^ mice. n = 7–9 mice per genotype. (F) Representative H and E-stained skin sections shown in E. (G) Analysis of epidermal thickness of 12–14-month-old mice. n = 4 male mice per genotype. (H) Representative H and E-stained of skin sections. (I) Kaplan–Meier curves depicting lesion-free survival of mice of the indicated genotypes. n = 30–48 mice per genotype. (J) Bliss independence analysis was performed to evaluate the potential synergistic effect between *Sirt2* and *Sod1* knockout on lesion development over time. (K) Liver tumor incidence of 12–14-month-old mice. n = 7–8 male mice per genotype. (L) Representative H and E-stained liver tumor sections. A.D.I, Data were calculated using log-rank test. C, E, G, Data are mean ± s.d. Two-tailed Student's t-test. K, Statistical significance was determined using a 2-tailed Fisher's exact test. J, Statistical significance was determined by permutation tests (1000 permutations). ∗*P <* 0.05, ∗∗*P* < 0.01, ∗∗∗*P* < 0.001, and ∗∗∗∗*P* < 0.0001. ns, not significant.

Lifespan analysis revealed that DKO mice had significantly shortened median lifespan (∼65 weeks) in comparison to either *Sirt2*^*−/−*^ or *Sod1*^*−/−*^ mice (Fig. 1A). Previous work has demonstrated that overexpressing *Sirt2* had no impact on mouse lifespan or healthspan [29], and our data also indicates that knocking out *Sirt2* had no significant effect on mouse lifespan at least during the observation window of at least 85 weeks (Fig. 1A), further suggesting that *Sirt2* might not be the critical factor regulating lifespan in a pathogen- and stress-free breeding environment. Additionally, *Sod1*^*−/−*^ mice exhibited mild lifespan shortening by ∼30 % compared to WT mice [9], and our data revealed that these mice had a median lifespan of over 83 weeks (Fig. 1A). Our data reveal that DKO mice had significantly shortened lifespan by at least 21.7 % in comparison to *Sod1*^*−/−*^ mice (Fig. 1A). In summary, these results reveal that the absence of *Sirt2* exacerbates progeroid features and further reduces lifespan in *Sod1*-deficient mice, suggesting a synergistic detrimental impact on organismal health.

Next, we set out to determine whether *Sirt2* deficiency could intensify the aging phenotypes associated with the absence of *Sod1* using these mice. Kyphosis, one of the primary aging-related phenotypes in both mice and humans, is characterized by an increased anterior curvature of the thoracic spine [30]. The analysis of kyphotic angle, which is the forward curvature of the spine and is used to quantify the degree of kyphosis, revealed that DKO mice had excessive curvature in comparison to WT, *Sirt2*^*−/−*^ or *Sod1*^*−/−*^ mice (Fig. 1B and C). The body weight analysis demonstrated a significant decrease in DKO mice compared to WT, *Sirt2*^*−/−*^ or *Sod1*^*−/−*^ mice (Fig. 1D). This could be partly attributed to the significant loss of subcutaneous fat in DKO mice (Fig. 1E and F). A distinct aging phenotype observed in the epidermis is the pronounced thickening of the cornified layer, as supported by a previous study [31]. In fact, the thickness of this layer was 3-fold greater in DKO mice compared to their wild type, *Sirt2*^*−/−*^, and *Sod1*^*−/−*^ counterparts (Fig. 1G and H).

Prior observations indicate that *Sod1*^*−/−*^ mice are susceptible to skin lesions [32]. Here we found that DKO mice exhibited early onset of mild dermatological pathologies (Fig. 1I). To assess the synergistic impact of *Sirt2* and *Sod1* deficiencies on lesion progression, Bliss independence analysis was conducted at intervals from 100 to 400 days. Initial time points (100 and 200 days) showed negligible synergy (scores: 0.062 and 0.055, respectively), not reaching statistical significance (*P* > 0.05). However, a robust synergistic effect was detected at 300 days with a score of 0.360 (95 % CI: 0.157–0.549, *P* = 0.0419), intensifying further at 400 days with a score of 0.474 (95 % CI: 0.229–0.714, *P* = 0.0241). The lesion incidence in DKO mice at 400 days (85.4 %) significantly surpassed the Bliss model-predicted rate (38.0 %), based on individual knockout effects (*Sirt2*^*−/−*^: 35.0 %, *Sod1*^*−/−*^: 4.5 %). Fisher's exact tests confirmed a significant correlation between genotype and lesion development at later stages (*P* < 0.001 for both 300 and 400 days) (Fig. 1J). Subsequent immunostaining assays utilizing an antibody against 8-oxoG disclosed heightened oxidative damage in DKO mice (Supplementary Fig. 1B). These results underscore a synergistic exacerbation of lesion progression attributed to an accumulation of oxidative stress caused by compounded *Sirt2* and *Sod1* deficiencies.

A previous report indicates that *Sod1* deficiency leads to the development of liver cancer at late age of mice (∼20 months) [9], which might attribute to persistent oxidative damage to DNA in livers. Given the fact that SIRT2 participates in the regulation of BER pathway, one would expect that knocking out *Sirt2* would accelerate the hepatocarcinogenesis in mice lacking *Sod1*. Indeed, DKO mice had significant increased liver tumor incidence at age of ∼12–14 months (Fig. 1K and L). Over 85 % of DKO mice developed liver tumor, while less than 20 % of *Sod1*^*−/−*^ mice had liver tumor and no hepatocarcinogenesis was observed in wild type or *Sirt2*^*−/−*^ mice (Fig. 1K and L).

Given the elevated hepatocarcinogenesis rate in DKO mice (Fig. 1K and L), we proceeded to investigate potential shifts in transcriptomic profiles associated with liver cancer. We established an expression profile based on the top 10 upregulated homologous genes in the livers of DKO mice (Supplementary Table 1), and compared their expression levels between human hepatocellular carcinoma and normal liver tissues in GEPIA2 database. Our analysis revealed a significant upregulation of the DKO-associated gene signature in human liver cancers relative to normal tissues (Supplementary Fig. 1C). Furthermore, survival analysis demonstrated that an elevated expression profile of this gene signature correlates with poorer prognosis in liver cancer patients (Supplementary Fig. 1D).

### 2.2 DKO mice exhibit greater transcriptomic alterations and an aging-associated transcriptome profile

**Fig. 2:**
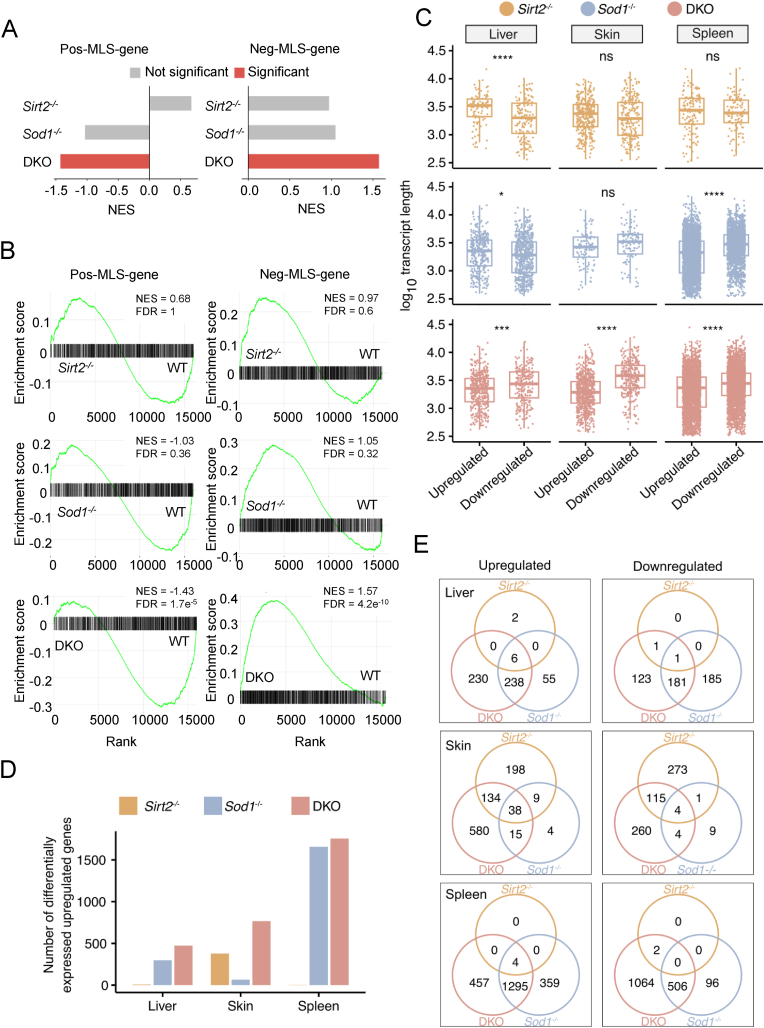
DKO mice exhibit an aging-associated transcriptome signature and undergo more transcriptomic changes compared to *Sirt2*^*−/−*^ and *Sod1*^−/−^ mice. (A) GSEA analysis demonstrates significant downregulation of pos-MLS genes and upregulation of neg-MLS genes in the skin of DKO mice. NES, normalized enrichment score. Significant represents False-discovery rate (FDR)-adjusted *P* < 0.05. (B) GSEA enrichment plots illustrate enrichment of pos-MLS genes among upregulated genes and neg-MLS genes among downregulated genes in the skin of DKO mice. (C) The transcript length distribution of differentially expressed genes in DKO mice resembles that of aging mice. Statistical significance was assessed using a two-tailed Wilcoxon test. ns, not significant; *∗P* < 0.05; *∗∗∗P* < 0.001; *∗∗∗∗P* < 0.0001. (D) The number of differentially expressed genes in DKO mice is significantly greater than that in *Sirt2*^*−/−*^ and *Sod1*^−/−^ mice. (E) Venn diagrams depict the overlap of differentially expressed genes in *Sirt2*^*−/−*^*, Sod1*^*−/−*^ and DKO mice.

To determine whether the transcriptome profiles reflect aging-associated changes, we performed RNA sequencing on the liver, skin, and spleen of female WT, *Sirt2*^*−/−*^, *Sod1*^*−/−*^, and DKO mice. Our transcriptomic analysis confirmed the genotype of different cohort of mice (Supplementary Fig. 2A). Principal component analysis showed that the samples segregated primarily by tissue rather than genotype (Supplementary Fig. 2B), while samples of different genotypes were clearly distinguishable within the same tissue (Supplementary Fig. 2C). First, we utilized two gene sets: pos-MLS, comprising genes positively correlated with rodent maximum lifespan, and neg-MLS, which contains genes negatively correlated with lifespan [33]. The pos-MLS gene set was downregulated in the skin and liver of DKO mice, while, conversely, the neg-MLS gene set was significantly upregulated in the skin and spleen of DKO mice compared to *Sirt2*^*−/−*^ and *Sod1*^*−/−*^ mice (Fig. 2A and B, and S2D). Thus, the double knockout of *Sirt2* and *Sod1* results in activation of negative-longevity genes and suppression of positive-longevity genes.

Aged mice also display length-associated transcriptome imbalances [34]. In DKO mice, the significantly upregulated transcripts were markedly shorter than the significantly downregulated transcripts across all the analyzed tissues. However, this transcriptome imbalance was not observed in *Sirt2*^*−/−*^ mice, and for *Sod1*^*−/−*^ mice, the difference was not observed in liver and skin, and mild in spleen (Fig. 2C).

In some organs, *Sirt2*^*−/−*^ or *Sod1*^*−/−*^ mice exhibited no significant transcriptome changes, whereas DKO mice displayed the highest number of differentially expressed genes across all examined organs. The number of differentially expressed genes in DKO mice exceeded the combined total of differentially expressed genes observed in *Sirt2*^*−/−*^ and *Sod1*^*−/−*^ mice (Fig. 2D). Moreover, the differentially expressed genes in DKO mice overlapped substantially with those of *Sirt2*^*−/−*^ and *Sod1*^*−/−*^ mice, including most of their respective differentially expressed genes (Fig. 2E).

These results indicate that DKO mice exhibit an aging- and negative-longevity-associated transcriptome profile, which is extensively perturbed, in comparison to *Sirt2*^*−/−*^ or *Sod1*^*−/−*^ mice.

### 2.3 The simultaneous depletion of *Sirt2* and *Sod1* accelerates accumulation of senescent cells in tissues and precipitates immune system dysregulation

**Fig. 3:**
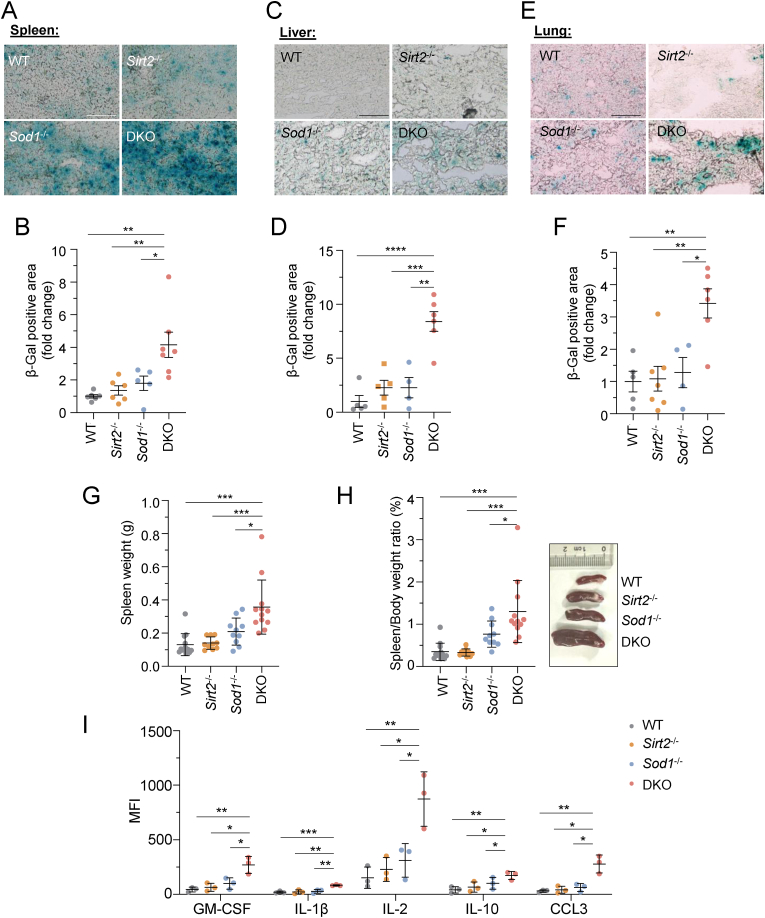
The simultaneous depletion of *Sirt2* and *Sod1* accelerates accumulation of senescent cells in tissues and precipitates immune system dysr**egulation.** (A–F) Senescence-associated β-galactosidase (SA-β-gal) staining analysis of spleens (A–B), livers (C–D), lungs (E–F) of 12–14-month-old mice. Representative SA-β-gal images are shown in (A, C, E), and the analysis of SA-β-gal positive area are shown in (B, D, F). n = 4–7 mice per genotype. Scale bar: 100 μm. (G) Spleen weight in WT, *Sirt2*^*−/−*^*, Sod1*^*−/−*^ and DKO mice. n = 10–13 mice per genotype. (H) Spleen/Body weight ratio in WT, *Sirt2*^*−/−*^*, Sod1*^*−/−*^ and DKO mice. n = 10–13 mice per genotype. (I) Median fluorescence intensity (MFI) was determined by flow cytometry to evaluate the level of cytokines in skin tissues of 12–14-month-old mice. n = 3 mice per genotype. B, D, F, Data are mean ± s.e.m. Mann-Whitney *U* test. G, H, I, Data are mean ± s.d. Two-tailed Student's t-test. *∗P* < 0.05; *∗∗∗P* < 0.001; *∗∗∗∗P* < 0.0001.

To quantify senescent cell burden, we performed the senescence-associated (β-galactosidase) SA-β-gal staining on tissue sections from DKO mice and their controls. Our results showed that the spleen, liver, and lung from DKO mice displayed signs of advanced aging compared to those from WT, *Sirt2*^*−/−*^, and *Sod1*^*−/−*^ mice (Fig. 3A–F). Additionally, the expression of p21, also a marker for senescent cells [[35], [36], [37]], was increased in the skin and lung tissues of DKO mice (Supplementary Fig. 3A–D), indicating a greater accumulation of senescent cells in these tissues from DKO mice.

We extended our investigation to determine if cells deficient in both genes would exhibit increased genomic instability. Despite our efforts, we were unable to successfully isolate DKO mouse fibroblasts, likely due to the vulnerability of DKO cells during *in vitro* culture. As an alternative approach, we isolated MEFs from WT mice and utilized siRNAs to knock down *Sirt2* and/or *Sod1*. We observed increased genomic instability, as measured by both comet assay and immunostaining with an anti-γH2AX antibody (Supplementary Fig. 3E–J), when compared to control cells or those with single gene knockdown of *Sirt2* or *Sod1*. Additionally, knocking down *Sirt2* and *Sod1* using siRNA greatly decreased the expression of Ki67, whose absence is also a molecular marker for cellular senescence (Supplementary Fig. 3K–L). Simultaneously knocking down *Sirt2* and *Sod1* increased the expression of p21 in the presence of H_2_O_2_ treatment in mouse cells (Supplementary Fig. 3M). These *in vitro* findings suggest that the absence of both *Sirt2* and *Sod1* contributes to enhanced DNA damage, genomic instability and cellular senescence at cellular level.

When analyzing the aging status of different tissues, we surprised observed enlarged spleens in DKO mice (Fig. 3G), and statistical analysis indicates this enlargement was significant (Fig. 3H). Since spleens are very critical immune organs, we therefore further examined cytokine profiling. Examination of key cytokines by FACS revealed that five cytokines up-regulated in DKO mice (Fig. 3I), further highlighting the radical changes in the matrix microenvironment in the remodeled spleen.

### 2.4 Transcriptome analysis suggests increased inflammation in DKO mice

**Fig. 4:**
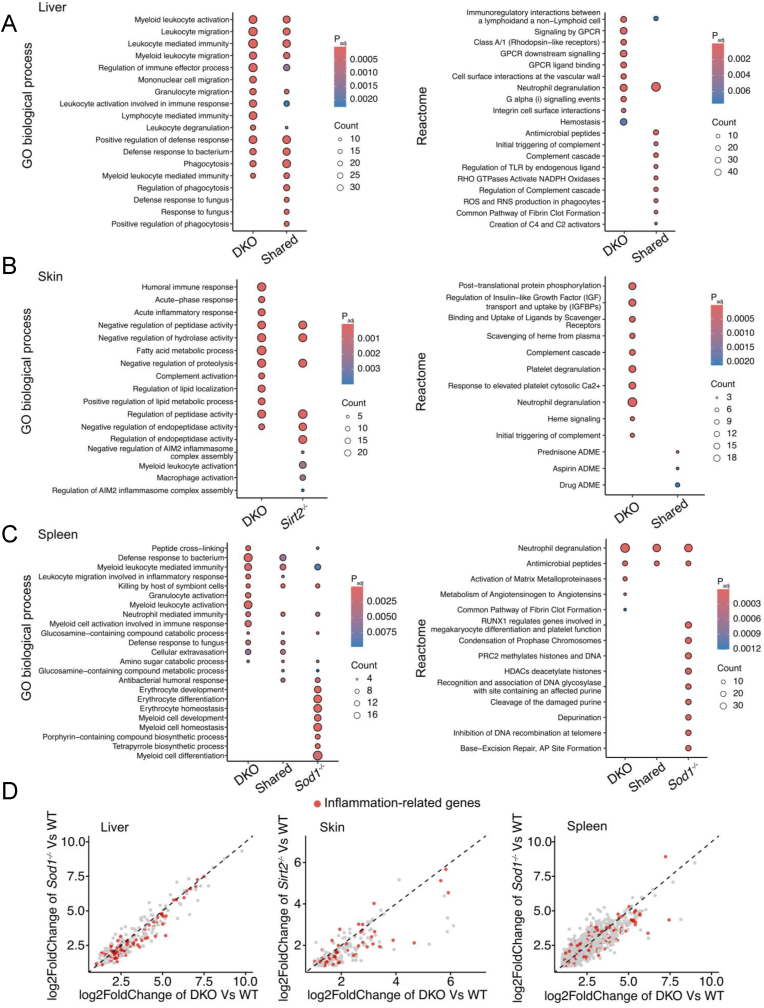
The transcriptome of DKO mice shows increased inflammation. (A) Pathway enrichment analysis of genes specifically upregulated in the liver of *Sod1*^*−/−*^ mice, specifically upregulated in the liver of DKO mice, and commonly upregulated in both. (B) Pathway enrichment analysis of genes that were specifically upregulated in the skin of *Sirt2*^*−/−*^ mice, specifically upregulated in the skin of DKO mice, and commonly upregulated in both. (C) Pathway enrichment analysis of genes specifically upregulated in the spleen of *Sod1*^*−/−*^ mice, specifically upregulated in the spleen of DKO mice, and commonly upregulated in both. (D) Scatter plots illustrate that inflammatory-associated genes are more highly upregulated in DKO mice compared to *Sirt2*^−/−^ and *Sod1*^−/−^ mice. Red dots indicate inflammatory-associated genes.

Since we observed the enlarged phenotype of the immune organ spleen, we speculated that there might be changes in immune-related pathways in DKO mice. We therefore conducted Gene Ontology (GO) term biological process and Reactome pathway enrichment analysis on the genes specifically upregulated in the *Sirt2*^*−/−*^, *Sod1*^*−/−*^, DKO mice, and commonly upregulated in them across all three organs. The results indicated a significant activation of inflammation-associated pathways in DKO mice, including enhanced activation and migration of myeloid cells and leukocytes, neutrophil activation and degranulation, regulation of Toll-like receptor (TLR) cascades, acute inflammatory responses, and immune complex activation, compared to the WT, *Sirt2*^*−/−*^, and *Sod1*^*−/−*^ mice. Furthermore, hemostasis, complement cascade activation and fibrin clot formation were upregulated in DKO mice, indicating increased tissue damage and bleeding, which likely contribute to the activation of these pathways. Additionally, senescence-associated pathways, such as the activation of matrix metalloproteinases, along with positive regulation of lipid metabolic process, were also upregulated in the spleen of DKO mice (Fig. 4A–C). Interestingly, in specific tissues such as the liver and skin, the genes uniquely upregulated in *Sirt2*^*−/−*^ or *Sod1*^*−/−*^ mice showed no enrichment in any particular pathway. This suggests that the transcriptome of DKO mice undergoes more extensive and systemic alterations (Fig. 4A and B).

Although certain immune-associated pathways were also upregulated in specific tissues of *Sirt2*^*−/−*^ or *Sod1*^*−/−*^ mice, we hypothesized that the inflammatory response is broader and more pronounced in DKO mice compared to *Sirt2*^*−/−*^ and *Sod1*^*−/−*^ mice. To further clarify this observation, we compared the fold change of commonly upregulated genes between DKO and *Sirt2*^*−/−*^ or *Sod1*^*−/−*^ mice. Immune-associated genes exhibited greater upregulation in DKO mice (Fig. 4D). In contrast, the pathways enriched by downregulated genes did not display unique commonalities specific to DKO mice (Fig. S4). In DKO mice, beyond the commonly upregulated genes, a substantial number of genes uniquely upregulated in DKO mice were also involved in immune-related pathways (Fig. 4A–C). These findings reveal a systemic activation of the immune profile in DKO samples, reflecting a coordinated response likely driven by the synergistic disruption of *Sod1* and *Sirt2*.

### 2.5 The DKO mouse transcriptome exhibits changes in the immune microenvironment

**Fig. 5:**
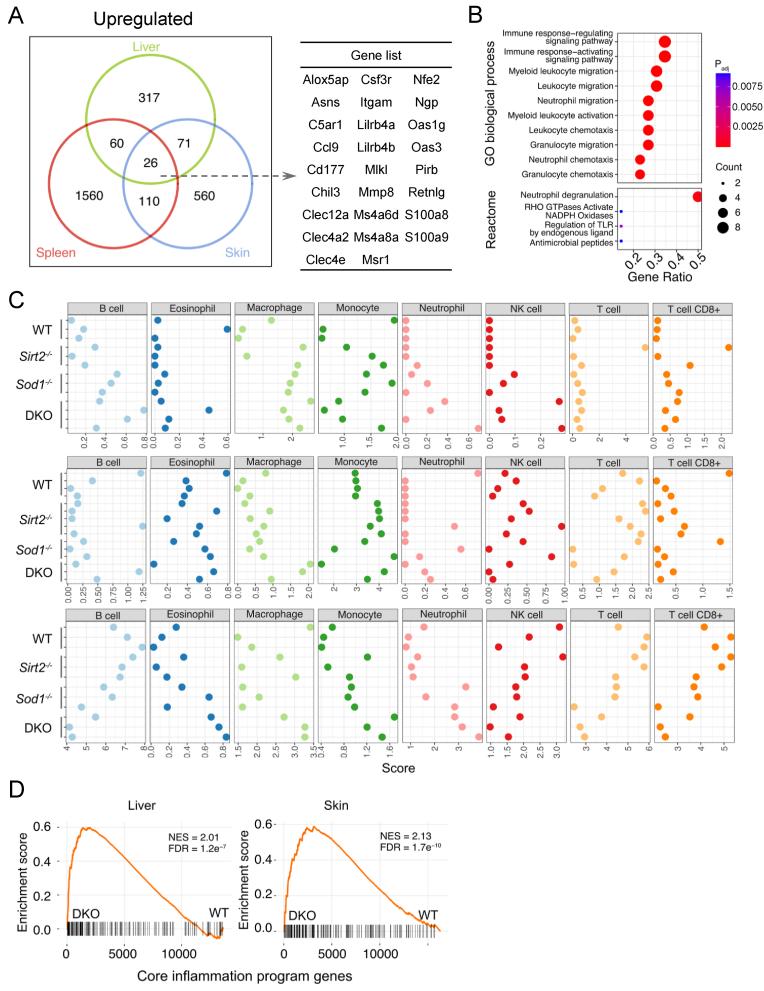
DKO mice transcriptome reveal the immune microenvironment changes. (A) Common up-regulated genes across all three tissues in DKO mice. (B) Pathway enrichment analysis of common up-regulated genes. (C) mMCP-counter scores of all samples demonstrate common changes in the immune microenvironment of DKO mice. (D) GSEA analysis demonstrates significant upregulation of core inflammation program gene set of neutrophils in the liver and skin of DKO mice. NES, normalized enrichment score.

We identified 26 commonly upregulated genes across all organs, constituting the core signature of DKO mice (Fig. 5A, S5A-B). We also confirmed the expression by quantitative PCR (Supplementary Fig. 5C). Interestingly, S100a8 and S100a9, which have recently been identified as novel aging biomarkers, were among these genes [38]. GO term biological process and Reactome pathway enrichment analysis revealed that these genes are primarily enriched in inflammatory pathways (Fig. 5B). Specifically, pathways such as neutrophil degranulation, migration, and neutrophil chemotaxis were highlighted, indicating activation and directed movement of neutrophils, key components of innate immunity. The enrichment of granulocyte migration and granulocyte chemotaxis pathways further underscores the increased mobilization of immune cells involved in inflammatory and immune responses. Additionally, the activation of RHO GTPases and NADPH oxidases, which are associated with the production of reactive oxygen species (ROS) and cytoskeletal rearrangements, was observed. These processes are critical for immune cell movement and function, particularly for macrophage activity [[39], [40], [41]]. Collectively, these findings underscore a coordinated and systemic activation of specific immune cell responses that are unique to the DKO condition across multiple tissues.

To further examine immune composition changes, we utilized mMCP-counter, a tool for estimating the immune cell composition in murine heterogeneous tissue using transcriptomic data, to compare *Sirt2*^*−/−*^, *Sod1*^*−/−*^ and DKO mice. The results demonstrated an increase in neutrophils and macrophages in DKO mice (Fig. 5C). Recent research has found that CD14^+^ macrophages and neutrophils are closely associated with the induction and progression of cytokine storms driven by TNF responses in a mouse sepsis model, significantly contributing to lethality [42]. We observed a significant upregulation of *Cd14* in the liver and skin of DKO mice (Supplementary Fig. 5D). Additionally, the core inflammation gene set associated with activated neutrophils [43] was also upregulated in the liver and skin of DKO mice (Fig. 5D). These findings suggest that the absence of *Sirt2* in *Sod1* knockout mice may alter the immune microenvironment, contributing to the aging phenotypes and shortened lifespan of DKO mice. This effect is likely driven by secreted immune factors and chronic inflammation induced by activated neutrophils and macrophages.

### 2.6 Overexpression of *Sirt2* rescues *Sod1*-deficiency mediated rise in genomic instability in MEFs

**Fig. 6:**
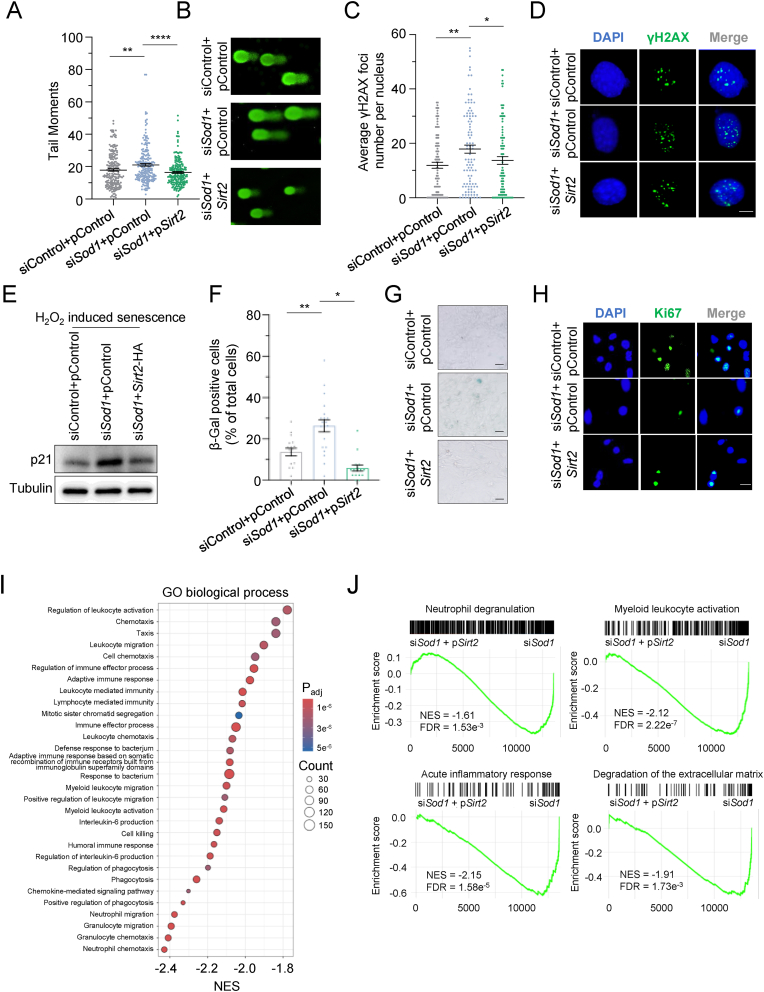
Overexpression of Sirt2 rescues *Sod1*-deficiency mediated-rise in genomic instabilit**y in MEFs.** (A) The genomic instability analysis in control MEFs, *Sod1*-depleted MEFs with or without *Sirt2* overexpressed using the comet assay. The cells were first transfected with si*Sod1*. Forty-eight hours later, the si*Sod1* and *Sirt2* were co-transfected into cells, and 16 h post-transfection, the cells were collected for genomic instability analysis. The tail moment was employed as the measure of genomic instability, and at least 100 cells were analyzed using the software CometScore. (B) Representative images of the comet assay. (C) Immunofluorescence analysis of γH2AX foci in control MEFs, *Sod1*-depleted MEFs with or without *Sirt2* overexpressed. The MEFs were first transfected with si*Sod1*. Forty-eight hours later, the si*Sod1* and *Sirt2* were co-transfected into MEFs, and 16 h post-transfection, the MEFs were collected for Immunofluorescence analysis. (D) Representative images of γH2AX foci. Scale bars, 10 μm. (E) Western blot analysis of p21 in control MEFs, *Sod1*-depleted MEFs with or without *Sirt2* overexpressed upon H_2_O_2_ treatment. The MEFs were first transfected with si*Sod1*. Forty-eight hours later, the si*Sod1* and *Sirt2* were co-transfected into MEFs, and 16 h post-transfection, different groups of MEFs were treated with H_2_O_2_ at a dosage of 200 μM. 3 days later, cells were harvested for protein extraction and Western blot analysis. (F) Senescence-associated β-galactosidase (SA-β-gal) staining analysis in control MEFs, *Sod1*-depleted MEFs with or without *Sirt2* overexpressed. Different groups of MEFs were treated with H_2_O_2_ at a dosage of 200 μM. 10 days later cells were harvested for SA-β-gal staining analysis. (G) Representative images of the SA-β-gal images. Scale bars, 50 μm. (H) Representative images of the Immunofluorescence analysis. Immunofluorescence analysis of the Ki67 positive cell analysis in control MEFs, *Sod1*-depleted MEFs with or without *Sirt2* overexpressed. Different groups of MEFs were treated with H_2_O_2_ at a dosage of 200 μM. 5 days later cells were harvested for Immunofluorescence analysis. Scale bars, 25 μm. (I) GSEA analysis of the full GO biological process collections, comparing *Sod1*-deficient MEFs overexpressing *Sirt2* with *Sod1*-deficient MEFs. (J) Representative enrichment plots for four pathways that are strongly up-regulated in *Sod1*^−/−^ or DKO mice—neutrophil degranulation, myeloid leukocyte activation, acute inflammatory response, and degradation of the extracellular matrix—illustrating their attenuation in *Sirt2* overexpressing *Sod1*-deficient MEFs. NES, normalized enrichment score. A, C, F, Data are mean ± s.e.m. Mann-Whitney *U* test. ∗*P* < 0.05, ∗∗*P* < 0.01 and ∗∗∗∗*P* < 0.0001.

To test whether *Sirt2* rescues *Sod1*-deficiency mediated rise in genomic instability, we performed comet assay and immunostaining with an anti-γH2AX antibody to analyze the changes in genomic stability by overexpressing *Sirt2* in *Sod1*-depleted MEFs. As expected, we observed that depleting *Sod1* using siRNA impaired genomic stability (Fig. 6A–D). Overexpressing *Sirt2* significantly rescued the *Sod1*-depletion mediated rise in genomic instability (Fig. 6A–D). Then, we determined whether *Sirt2* rescues *Sod1*-deficiency mediated rise in cellular senescence, we examined the level of p21, pRPS6, Ki67 and the activity of SA-β-gal in mouse cells. We found that knocking down *Sod1* using siRNA greatly promoted the expression of p21 in the absence or presence of X-ray irradiation or H_2_O_2_ treatment cells, while overexpressing *Sirt2* attenuated *Sod1*-deficiency induced increase in p21 level (Fig. 6E, Supplementary Fig. 6A–C). Overexpressing *Sirt2* also inhibited the SA-β-galactosidase activity, increased the level of pRPS6 and promoted the expression of Ki67 in H_2_O_2_ induced senescent MEFs (Fig. 6F–H, Supplementary Fig. 6D–E). To delineate the transcriptional impact of *Sirt2* on *Sod1*-deficient MEFs, we performed bulk RNA-seq on *Sod1-*deficient MEF cells with and without *Sirt2* overexpression. GSEA analysis against the complete Reactome and GO biological process libraries revealed that *Sirt2* overexpression significantly attenuated immune- and senescence-associated pathways (Fig. 6I, Supplementary Fig. 6F). In particular, pathways including neutrophil degranulation, myeloid leukocyte activation, acute inflammatory response, and degradation of the extracellular matrix, which were significantly enriched in *Sod1*^*−/−*^ or DKO mice, were markedly reduced in *Sod1*-deficient MEFs after *Sirt2* overexpression (Fig. 6J). These findings indicate that targeting SIRT2 could be a promising strategy to counteract DNA damage, inflammation and cellular senescence triggered by free radicals.

## 3 Discussion

The free radical theory of aging, which posits that the accumulation of ROS contributes to aging, has been a subject of debate in the scientific community. A primary contention is the lack of robust evidence demonstrating that ROS accumulation directly leads to premature aging. This may be attributed to the fact that cells possess highly efficient DNA repair mechanisms that prevent excessive accumulation of ROS. To further substantiate the role of ROS in aging, it is necessary to disable the DNA repair pathways, thereby allowing for the accumulation of ROS-induced DNA damage.

In our study, we have provided novel evidence supporting the free radical theory of aging by showing that the knockout of *Sirt2*, a regulator of 10.13039/100006206BER pathway, significantly accelerates aging in *Sod1* knockout mice. However, since SIRT2 is involved in the regulation of multiple pathways, to further confirm that the observed aging is indeed due to unrepaired ROS-induced DNA damage, we propose the targeted knockout of specific non-essential BER pathway genes, such as *Ogg1*, a DNA glycosylase with no apparent phenotype when knocked out in mice. Crossing these *Ogg1* knockout mice with *Sod1* knockout mice could provide additional evidence for the role of ROS in aging.

Furthermore, *Sod1* knockout mice exhibit mild premature aging phenotypes and mutations in the *Sod1* are associated with certain neurodegenerative diseases [44,45]. Our study found overexpressing *Sirt2* rescued *Sod1*-deficiency mediated cellular senescence. Although cellular senescence can serve beneficial roles, such as tumor suppression and wound healing, chronic accumulation of senescent cells is widely recognized as a contributor to age-related functional decline [36, 37]. Targeting the activation of *Sirt2* expression, for instance, by generating a transgenic mouse overexpressing *Sirt2* and crossing it with *Sod1* knockout mice, could test whether this approach alleviates the premature aging and age-related neurodegenerative disease phenotypes observed in *Sod1* knockout mice. This would offer new insights and strategies for the development of novel lifespan-extending drugs.

Additionally, directly activating the BER pathway by overexpressing multiple factors within the pathway, such as *Ape1*, *Xrcc1*, and *Pol β*, and crossing these transgenic mice with *Sod1* knockout mice, could explore whether activating the BER pathway can mitigate aging and related diseases caused by *Sod1* deficiency. This approach would provide new perspectives and directions in the field of aging and ROS, as well as in the broader area of DNA repair.

Our study analyzed the transcriptomic effects of *Sirt2* and *Sod1* DKO in mice, revealing more pronounced aging-like changes compared to single knockouts. DKO mice showed increased inflammatory responses, with upregulation of pro-inflammatory genes and pathways, aligning with the "inflammaging" concept [46,47]. A set of 26 upregulated genes, including *S100a8* and *S100a9* discovered by a previous report [38], indicated potential as aging biomarkers and participants in inflammatory and age-related diseases. The activation of RHO GTPases and NADPH oxidases pointed to heightened ROS production and cytoskeletal rearrangements related pathways changes due to the loss of *Sod1* and *Sirt2*. Immune cell analysis confirmed increased neutrophils and macrophages, consistent with the upregulated immune pathways, potentially driving tissue damage and aging. The simultaneous loss of *Sirt2* and *Sod1* in DKO mice accelerated age-associated immune changes, contributing to inflammation, tissue damage, and aging. These findings suggest that targeting oxidative stress and immune dynamics could combat age-related inflammation and its consequences.

## 4 Materials and methods

### 4.1 Mice

Mice used in this study included WT, *Sirt2*^*−/−*^*, Sod1*^*−/−*^, and *Sirt2*^*−/−*^ *Sod1*^*−/−*^ mice in a genetic background of C57BL/6J. All mice were housed in SPF facilities of the Animal Resource Center at Tongji University. All procedures were approved by the Tongji University Animal Care and Use Committee (TJAB04022106). *Sirt2* knockout mice (stock #012772) were obtained from JAX lab. *Sod1* knockout mice were generated with conventional CRISPR-Cas9 technique. *Sirt2*^*+/−*^*, Sod1*^*+/−*^ mice were crossed to obtain WT, *Sirt2*^*−/−*^*, Sod1*^*−/−*^, and *Sirt2*^*−/−*^ *Sod1*^*−/−*^ mice for further lifespan and other types of phenotypical analysis in this study. Genomic DNA was extracted from mouse tails using lysis buffer (50 mM pH 8.0 Tris–HCl, 100 mM EDTA, 1 % SDS, 20 mg/ml Proteinase K) for genotyping. The primers for PCR involved in this process include the following: 5′-GACTGGAAGTGATCAAAGCTC-3′, 5′-CAGGGTCTCACGAGTCTCATG-3′, 5′-TCA AAT CTGGCCAGAACTTATG-3’ (*Sirt2*); 5′-AACTTTCTCAGTCCGCACGCT-3′, 5′-GCAGCAGCCCCAGAAGGATAA-3′, 5′-ATGCTGGCCTTCAGTTAATCC-3’ (*Sod1*).

### 4.2 H&E staining, analysis of subcutaneous adipose tissue and epidermal thickness

The subcutaneous (s.c.) adipose tissue and epidermal thickness of WT, *Sirt2*^*−/−*^*, Sod1*^*−/−*^, and *Sirt2*^*−/−*^*Sod1*^*−/−*^ mice was assessed using standard H&E staining on skin histology sections (Beyotime, C0105). Images were captured at 10 × magnification across the entire section using an OLYMPUS BX53 microscope. In these images, the thickness of the s.c. adipose tissue was measured at 12 distinct points per section using the AxioVision 4.8 software. The average of the 12 measurements was then calculated to determine the adipose thickness of each mouse.

### 4.3 Immunofluorescence staining

Skin tissues were formalin-fixed and paraffin-embedded. Antigen retrieval was conducted in citrate buffer (pH = 6) for skin sections. Primary antibody staining was performed using anti-8-oxo-dG (4354-mc-050, Trevigen R&D), followed by counterstaining with DAPI.

For cellular immunofluorescence, cells transfected with the indicated siRNA were plated on coverslips in 12-well plates. Twenty hours post-transfection, cells were rinsed with cold PBS and fixed with 4 % paraformaldehyde (PFA) for 15 min at room temperature. Then, the cells were permeabilized with 0.25 % Triton X-100 for 15 min and blocked with 2 % bovine serum albumin (BSA) for 1 h at room temperature. Cells were then incubated with a γH2AX antibody overnight at 4 °C, followed by incubation with a secondary antibody for 1 h at room temperature in the dark. Cells were mounted in mounting medium containing DAPI, and images were acquired with a Nikon laser scanning confocal microscope.

### 4.4 SA β-gal staining

Tissues were snap-frozen in OCT compound, fixed in a solution of 2 % formaldehyde and 0.2 % glutaraldehyde in PBS for 5 min at room temperature, and washed twice with PBS. Staining solution (1 mg/mL X-gal in dimethylformamide, 40 mM citric acid/sodium phosphate buffer, 5 mM potassium ferrocyanide, 5 mM potassium ferricyanide, 150 mM sodium chloride, and 2 mM magnesium chloride) was applied, and the tissues were incubated for 24 h at 37 °C. Images of the stained tissues were taken using a bright-field microscope.

### 4.5 Protein preparation from tissues and Western blot

Western blot analysis was performed on total protein extracts from brain, skin and lung tissues, which were homogenized in RIPA buffer (20 mM Tris-HCl, pH 7.6, 150 mM NaCl, 1 % NP-40, 1 % sodium deoxycholate, 0.1 % SDS, 1 mM PMSF, 5 mM sodium butyrate and 2 mM sodium vanadate) containing protease and phosphatase inhibitors. Lysates were mixed with 2 × sample buffer, boiled for 10 min, and subjected for Western blot analysis using antibodies including anti-Sirt2 (09843, Sigma-Aldrich), anti-Sod1 (A12537, Abconal), anti-p21 (ab109520, Abcam), anti-Tubulin (Cat# AP0064, Bioworld).

### 4.6 Alkaline comet assay

Cells transfected with the indicated siRNA were seeded on coverslips in 6-well plates. Twenty hours post-transfection, cells were collected, resuspended in PBS and diluted to 3 × 10^5^ cells per ml before the comet assay was performed. The detailed procedure is described in the manufacturer's instructions (Trevigen, Cat. # 4250-050-K). Tail moments were used to quantify the amount of DNA damage using CometScore software (casplab_1.2.3b2).

### 4.7 Multi-parameter flow cytometry

Total protein was extracted from skin tissues and stained with antibodies against mouse GM-CSF, IL-1β, IL-2, IL-10, CCL3 (RK04397, ABconal), and analyzed using Multi-index flow analyzer (ABclonal, ABplex-100).

### 4.8 RNA isolation and RNA sequencing

Mice that showed any macroscopic lesions at necropsy on histological screening were excluded a priori. Total RNA was extracted from tissue from tumor-free samples using TRIzol® Reagent (Invitrogen, 15596026), following the manufacturer's protocol. Subsequent RNA purification, reverse transcription, library construction, and sequencing services were provided by Shanghai Majorbio Bio-pharm Biotechnology Co., Ltd. in Shanghai, China, adhering to Illumina's guidelines (Illumina, San Diego, CA).

For RNA-seq, a transcriptome library was prepared using the Illumina® Stranded mRNA Prep, Ligation kit (Illumina, San Diego, CA), starting with 1 μg of total RNA. mRNA was enriched through polyA selection with oligo(dT) beads, followed by fragmentation using a fragmentation buffer. Double-stranded cDNA synthesis was carried out using the SuperScript double-stranded cDNA synthesis kit (Invitrogen, CA) and random hexamer primers supplied by Illumina. The cDNA underwent end-repair, phosphorylation, and the addition of 'A' bases, following Illumina's library construction protocol.

Libraries were size-selected to target cDNA fragments of 300 bp using 2 % Low Range Ultra Agarose, and then amplified via PCR with Phusion DNA polymerase (NEB) for 15 cycles. Quantified using a Qubit 4.0 fluorometer, the paired-end RNA-seq libraries were sequenced on a NovaSeq X Plus sequencer, generating 2 × 150bp read lengths. RNA-seq analysis was carried out using three biological replicates for each tissue.

### 4.9 RNA extraction and real-time quantitative PCR

Total RNA was extracted using an RNAsimple Total RNA Kit (TIANGEN) and subsequently reverse transcribed into cDNA using the TransScript II Reverse Transcriptase kit (TRANS). Briefly, qRT–PCR of *S100-a9*, *Prib*, *Ms4a8a* and *Ccr1* genes was performed. Real-time PCR was performed with FastStart DNA Master SYBR Green Mix (Roche, 4913914001) on a ViiA 7 Real-Time PCR system (Applied Biosystems). The average threshold cycle (Ct) of quadruplicate reactions was determined, and expression was analyzed by the ΔΔCt method. The relative expression levels were normalized to the level of *Gapdh*. The primers used to amplify s100-a9 were as follows: *s100-a9*-mRNA-F, 5′-GCCAACAAAGCACCTTCTCA-3′; *s100-a9*-mRNA-R, 5′-TGTCAGGGTGTCCTTCCTTC-3′; Prib-mRNA-F, 5′-AACAATCAGGCTGCCGAATC-3′; *Prib*-mRNA-R, 5′-CTGGGAGAGAGGAGATGCAG-3′; *Ms4a8a*-mRNA-F, 5′-CGCCCAACAGTTATCCTGTG-3′; *Ms4a8a*-mRNA-R, 5′-GGACTTGAGGCTGATTGCTG-3′; *Ccr1*-mRNA-F, 5′-ACCTGTAGCCCTCATTTCCC-3′; *Ccr1*-mRNA-R, 5′-CTCACTGGGTCTTCTGAGCA-3′.

### 4.10 RNA-seq analysis

Total RNA was isolated from liver, skin, and spleen of 12–14-month-old mice. Quality control of RNA-seq data was performed using FastQC v0.11.9. Adapters and low-quality reads, and reads with length <20 bp were removed using Cutadapt v2.8. Quantification of samples was conducted at both gene and transcript levels against the mouse GRCm39 cDNA reference using Salmon v1.10.3 with default parameters. Only genes with read counts >10 in at least 3 samples were retained for downstream analysis. Differentially expressed genes (DEGs) were identified using DESeq2 v1.16.1, with thresholds set at log_2_ fold change >1 or < −1 and adjusted p-value (padj) < 0.05. Genes meeting these criteria were defined as DEGs.

### 4.11 Gene set enrichment analysis (GSEA) and pathway over-representation analysis

GSEA was performed on a pre-ranked gene list, ranked by the formula: -log10 [padj] × (fold change)/abs(fold change), using fgsea v1.32.0 with parameters minSize = 15 and maxSize = 500. Gene sets with a FDR <0.05 were considered significant. Enrichment analysis for GO Biological Processes and Reactome pathways was conducted using the R package clusterProfiler v4.12.2. P-values were adjusted using the Benjamini–Hochberg correction, with significance thresholds set at p-value <0.01 and adjusted p-value <0.05.

### 4.12 Immune microenvironment evaluation

Changes in the immune microenvironment were assessed based on gene expression profiles of all samples using mMCP-counter, implemented through the R package immunedeconv v2.1.3.

#### 4.12.1 Bioinformatics analysis limitations

Several limitations should be acknowledged regarding our RNA-seq analysis approach. First, the relatively modest sample size may limit statistical power for detecting genes with smaller effect sizes, potentially leading to false negatives despite our stringent statistical thresholds. Second, while the Benjamini-Hochberg correction effectively controls the false discovery rate, this conservative approach may increase the risk of Type II errors, potentially overlooking biologically relevant genes with moderate expression changes. Third, our differential expression analysis relies on predefined fold change and adjusted p-value thresholds (abs(log_2_ fold change) > 1 and padj <0.05), which, while commonly used, are somewhat arbitrary and may not capture all biologically meaningful changes. Fourth, gene set enrichment analyses are inherently dependent on the completeness and accuracy of existing pathway annotations, which may introduce bias toward well-characterized biological processes while potentially missing novel or poorly annotated pathways. Fifth, regarding immune microenvironment evaluation, the mMCP-counter algorithm infers cell-type proportions based on reference gene signatures derived primarily from younger, healthy mouse tissues. The aged mouse tissues analyzed here may deviate from these reference signatures, potentially reducing absolute accuracy. Consequently, results from this method were interpreted cautiously and primarily considered in relative terms rather than absolute cell-type proportions.

## CRediT authorship contribution statement

**Anke Geng:** Conceptualization, Data curation, Writing – original draft, Writing – review & editing. **Xiaona Wang:** Data curation, Formal analysis. **Zhenkai Wu:** Software. **Zhihao Liu:** Formal analysis, Methodology. **Xiao Huang:** Formal analysis. **Xiyue Wang:** Data curation. **Xiaoxiang Sun:** Data curation. **Yingjie Wang:** Data curation. **Jiayu Chen:** Resources. **Ying Jiang:** Conceptualization, Writing – original draft, Writing – review & editing. **Huanyin Tang:** Conceptualization, Data curation, Writing – original draft, Writing – review & editing. **Zhiyong Mao:** Conceptualization, Data curation, Writing – original draft, Writing – review & editing.

## Data and materials availability

All data supporting the conclusions in this manuscript can be found in the main text or the supplementary materials. The RNA-seq data generated in this study have been deposited in the National Genomics Data Center (NGDC) and can be accessed using the provided accession number CRA021511.

## Funding

This work was supported by the National Key R&D Program of China (2022YFA1103703 and 2021YFA1102003 to Z.M.), the 10.13039/501100001809National Natural Science Foundation of China (Grant Nos. 32171288 and 32471341 to Y.J., 32100605 to A.G.), and the 10.13039/501100002858China Postdoctoral Science Foundation (2023M732662 to H.T.).

## Declaration of competing interest

The authors declare that they have no known competing financial interests or personal relationships that could have appeared to influence the work reported in this paper.
