# Supplementary material for: Sirt2 ablation exacerbates Sod1 knockout-induced progeroid phenotype in mice
Source: Redox Biol. 2025 Jul 15;85:103770. doi: 10.1016/j.redox.2025.103770 (PMC12301975; doi:10.1016/j.redox.2025.103770)
Supplement: Multimedia component 1 [file mmc1.docx]

Fig. S1. Related to Fig. 1. (A) Western blot analysis of brain extracts from wild-type (WT), *Sirt2^-/-^, Sod1^-^*^/-^ and DKO mice. (B) Representative images of skin sections from 12-14-month-old mice of the indicated genotypes, stained with an anti-8-oxoG antibody and DAPI (DNA stain). n = 3 mice. Scale bars, 50 μm. (C) The boxplot presents the expression profile of a gene signature composed of the top 10 homologous genes upregulated in DKO mouse livers, highlighting differences between human liver cancer tissues (red) and normal liver tissues (blue). *****P* < 0.0001. (D) Based on the expression of this genetic feature, liver cancer patients are stratified into a high-expression group (red) and a low-expression group (blue), with the high-expression group demonstrating a poorer prognosis.

Fig. S2. Related to Fig. 2. (A) Representative IGV plots showing read coverage at the *Sirt2* and *Sod1* loci in *Sirt2^-/-^*, *Sod1^-^*^/-^ and DKO mice. (B) PCA analysis of all liver, skin and spleen samples from WT, *Sirt2^-/-^*, *Sod1^-^*^/-^ and DKO mice. (C) PCA analysis was performed separately for liver, skin, and spleen samples from WT, *Sirt2^-/-^*, *Sod1^-^*^/-^and DKO mice. (D) GSEA analysis indicates significant downregulation of pro-MLS genes and upregulation of neg-MLS genes in DKO mice. Significant represents False-discovery rate (FDR)-adjusted *P* < 0.05.

Fig. S3. Related to Fig. 3. (A-D) Western blot analysis of skin (A-B), lung (C-D) extracts from 12-14-month-old mice Representative pictures are shown with three pairs per genotype in (A, C), and the quantification of p21 expression is shown in (B, D). n=5-10 mice per genotype. (E) Western blot analysis of *Sirt2* in control and *Sirt2*-knockdown cells. (F) Western blot analysis of *Sod1* in control and *Sod1*-knockdown cells. (G) The genomic instability analysis in control, *Sirt2*-depleted, *Sod1*-depleted and double gene-depleted MEFs using the comet assay. The tail moment was employed as the measure of genomic instability, and at least 100 cells were analyzed using the software CometScore. (H) Representative images of the comet assay. (I) Immunofluorescence analysis of γH2AX foci in control, *Sirt2*-depleted, *Sod1*-depleted and double gene-depleted MEFs. (J) Representative images of γH2AX foci. Scale bars, 10 μm. (K) Immunofluorescence analysis of the Ki67 positive cell analysis in control, *Sirt2*-depleted, *Sod1*-depleted and double gene-depleted cells. (L) Representative images of the Immunofluorescence analysis. Scale bars, 20 μm. (M) Western blot analysis of p21 in control MEFs, *Sirt2*-depleted, *Sod1*-depleted and double gene-depleted MEFs upon H_2_O_2_ treatment. The MEFs were transfected with si*Sirt2* and si*Sod1*. Forty-eight hours later, the si*Sirt2* and si*Sod1* were transfected into MEFs, and 16 h post-transfection, different groups of MEFs were treated with H_2_O_2_ at a dosage of 200 μM. 3 days later, cells were harvested for protein extraction and Western blot analysis. B, D, Data are mean ± s.d. Two-tailed Student’s t-test. G, I, K, Data are mean ± s.e.m. Mann-Whitney U test. **P* < 0.05, ***P* < 0.01, ****P* < 0.001, and *****P* < 0.0001.

Fig. S4. Related to Fig. 4. (A) Pathway enrichment analysis of genes specifically downregulated in the liver of *Sod1*^-/-^ mice, specifically downregulated in the liver of DKO mice, and commonly downregulated in both. (B) Pathway enrichment analysis of genes that were specifically downregulated in the skin of *Sirt2*^-/-^ mice, specifically downregulated in the skin of DKO mice, and commonly downregulated in both. (C) Pathway enrichment analysis of genes specifically downregulated in the spleen of *Sod1*^-/-^ mice, specifically downregulated in the spleen of DKO mice, and commonly downregulated in both.

Fig. S5. Related to Fig. 5. (A) The Venn diagram illustrates that no common downregulated genes, except for *Sirt2* and *Sod1*, are shared across all tissues of DKO mice. (B) Boxplots display the expression levels of the 26 common upregulated genes. (C) Quantitative PCR confirmed the expression of the common upregulated genes. (D) Cd14 was significant upregulated in liver and skin of DKO mice. Statistical significance was accessed by two-tailed Student’s t test. **P* < 0.05; ***P* < 0.01; ****P* < 0.001; *****P* < 0.0001.

Fig. S6. Related to Fig. 6. (A) Western blot analysis of *Sirt2* overexpression in MEFs was performed. (B) Western blot analysis of p21 in control MEFs, *Sod1*-depleted MEFs with or without *Sirt2* overexpressed. (C) Western blot analysis of p21 in control MEFs, *Sod1*-depleted MEFs with or without *Sirt2* overexpressed upon X-ray irradiation. The MEFs were first transfected with si*Sod1*. Forty-eight hours later, the si*Sod1* and *Sirt2* were co-transfected into MEFs, and 16 h post-transfection, different groups of MEFs were irradiated with X-ray at a dosage of 10 Gy. 5 days later, cells were harvested for protein extraction and western blot analysis. (D) Western blot analysis of pRPS6 in control MEFs, *Sod1*-depleted MEFs with or without *Sirt2* overexpressed upon H_2_O_2_ treatment, **using the same method as in Figure 6E**.**** (E) Immunofluorescence analysis of the Ki67 positive cell analysis in control MEFs, *Sod1*-depleted MEFs with or without *Sirt2* overexpressed. Different groups of MEFs were treated with H_2_O_2_ at a dosage of 200 μM. 5 days later cells were harvested for immunofluorescence analysis. (F) GSEA analysis of the full Reactome collections, comparing *Sod1*-deficient MEFs over-expressing *Sirt2* with *Sod1*-deficient MEFs. NES, normalized enrichment score. Data are mean ± s.e.m. Mann-Whitney U test. **P* < 0.05, ***P* < 0.01.

Table S1. Top 10 upregulated homologous genes in the livers of DKO mice.
